# Utilisation of mobile apps in neurological rehabilitation practice among occupational therapists in India: a cross-sectional survey

**DOI:** 10.1186/s12913-026-14098-w

**Published:** 2026-02-03

**Authors:** S. Gokul Prasath, Guruprasad Vijayasarathi, Shashank Mehrotra

**Affiliations:** 1 Department of Occupational Therapy, Manipal College of Health Professions, Manipal Academy of Higher Education, Manipal, India; 2 Present Address: Clinical Research, National Centre for Assistive Health Technologies (NCAHT), TTK Center for Rehabilitation Research and Device Development, Machine Design Section, Indian Institute of Technology Madras, Chennai, India; 3 Occupational Therapy Program, School of Rehabilitation and Medical Sciences, College of Health Sciences, University of Nizwa, Nizwa, Sultanate of Oman; 4 Center for Comprehensive Stroke Rehabilitation and Research (CCSRR), Manipal College of Health Professions, Manipal Academy of Higher Education, Manipal, India

**Keywords:** Mobile apps, Occupational therapists, Neurological rehabilitation, Barriers, Facilitators, Human Activity Assistive Technology (HAAT)

## Abstract

**Background:**

There is an increasing trend in the utilisation of mobile applications (apps) and smartphones in rehabilitation. This study aims to explore the utilisation of mobile apps among Occupational Therapists (OTs) in neurological rehabilitation practice in India.

**Methods:**

We employed a cross-sectional survey design, convenience sampling, and snowball sampling methods to identify the study participants. An online questionnaire comprising 28 questions was sent to the participants. Quantitative data were analysed using Jamovi V2.3.32, and the qualitative data (open-ended questions) were analysed using content analysis.

**Results:**

The study revealed that 42% of OTs working in neurological rehabilitation practice in India utilised mobile apps in clinical practice. Most participants reported using such apps as an intervention modality (80%) rather than an assessment tool (40%). Client engagement was the most common reason for using apps, and the effectiveness of apps is perceived to be the same or better than that of traditional methods. Despite the presence of facilitators, such as client engagement and active participation, barriers, such as limited knowledge and technological unfamiliarity, were identified.

**Conclusion:**

This study highlights the significant proportion of occupational therapists who incorporate mobile apps into neurological practice, along with their perceived barriers and facilitators. Addressing such barriers through user-centric and practitioner involvement can maximise the potential of app-based practice in neurological rehabilitation by occupational therapists.

**Supplementary Information:**

The online version contains supplementary material available at 10.1186/s12913-026-14098-w.

## Strengths and limitations of this study

This study investigated the use of mobile apps by occupational therapists in neurological rehabilitation practice in India, offering a comprehensive examination of a relatively underexplored field.

This study utilised a structured approach by employing the Human Activity Assistive Technology (HAAT) model for analysing facilitators, barriers, and future recommendations related to the use of mobile apps, allowing for a holistic understanding of app integration into occupational therapy practice.

The survey's limited sample size may not reflect the entirety of occupational therapists working with clients experiencing neurological conditions in India.

Moreover, it is worth noting that the survey tool was tailored for this study and lacked psychometric properties.

**Supplementary Information:**

The online version contains supplementary material available at 10.1186/s12913-026-14098-w.

## Introduction

Smartphones have become part of everyday life. With smartphones, sharing thoughts and transferring information, more efficient ways of doing daily tasks are easily achieved [1]. According to Taylor [2], the global number of smartphone users reached 6.8 billion in 2023. In India, there were approximately 748 million smartphone users in 2020, and this figure is projected to surpass 1.5 billion by 2040 [3]. This expansion highlights the increasing significance of mobile applications in the Indian healthcare system, where digital health adoption has been a key focus of national initiatives, such as the National Digital Health Mission (NDHM) [4].

A mobile application, or app, is a software program designed to run on smartphones and tablet platforms [4, 5], particularly on prominent operating systems, with more than 3.5 million Android apps in Google Play and 1.6 million iOS apps in the Apple App Store as of 2022 [6].

Smartphones and mobile applications are increasingly used in clinical practice, serving purposes ranging from quick reference checks to more advanced, interactive clinical tools. They support patient monitoring, data collection, telerehabilitation, and documentation, benefiting clinicians, researchers, and patients [7]. Globally, rapid growth is seen in mobile apps dedicated to health and wellness, including rehabilitation, chronic disease management, and assistive technologies [8–12].

Smartphones and tablets are increasingly recognised as assistive technology devices due to their accessibility features, portability, and ability to support individuals with diverse impairments [13]. These devices offer built-in tools, such as screen readers, voice commands, magnifiers, and speech-to-text apps, that empower users with visual, motor, or cognitive challenges to communicate and navigate their environments independently [13, 14]. Their affordability and widespread availability make them especially valuable in low- and middle-income countries like India, where access to traditional technologies may be limited [13]. Moreover, apps can transform smartphones into picture boards, navigation aids, or health trackers, supporting personalised interventions across a range of disabilities [14, 15]. As mobile technology continues to evolve, its role in inclusive healthcare and neurorehabilitation is also growing [15].

A national survey from the United States reported that approximately 71.9% of occupational therapy practitioners use mobile applications in their clinical practice [16]. These apps support individuals with neurological conditions such as stroke, traumatic brain injury, and spinal cord injury by enhancing engagement, promoting accessibility to rehabilitation exercises and self-management tools, and supporting remote monitoring of functional progress [17].

Neurorehabilitation primarily aims to achieve the highest level of functional autonomy, encompassing physical, cognitive, and behavioural independence [18], aligning with the Occupational Therapy Practice Framework [19], which emphasises meaningful participation across self-care, productivity, and leisure occupations [20].

Occupational therapists are increasingly involved in app development [21]. Examples include Dexteria and Aid for Decision Making in Occupational Choice (ADOC). The Dexteria app has been shown to improve dexterity, eye-hand coordination, engagement, motivation, and learning, thereby encouraging independence in daily tasks [22]. Meanwhile, Tomori et al. [23] demonstrated that ADOC supports shared decision-making and occupation-based goal setting. The existing literature suggests that occupational therapists often find such apps valuable for assessment and intervention, particularly in paediatric practice, and sometimes consider them more effective than conventional methods [16, 24, 25]. Although app use is often reported to be higher in paediatrics, evidence shows that cognitive and neuromusculoskeletal functions remain among the most frequently targeted client factors in practice [16]. Studies indicate that many commonly used clinical apps support cognitive training, motor function, and functional task performance [16, 17]. These domains are central to neurorehabilitation, further emphasising the relevance of app-based tools in this area [18].

Despite the increasing availability of mobile apps and India’s rapidly expanding digital health ecosystem, there is limited evidence on how occupational therapists in India integrate mobile apps specifically within neurological rehabilitation practice. Neurological disorders are placing an increasing public-health burden in India; for example, in 2019, non-communicable neurological conditions such as stroke, headache disorders, and epilepsy contributed nearly 8.2% of the country’s total disability-adjusted life years (DALYs), with stroke alone accounting for 37.9% of neurological DALYs [26]. At the same time, smartphone penetration and digital-health adoption are expanding rapidly in India [27], creating a timely opportunity to integrate mobile applications into neurorehabilitation practice. Investigating how occupational therapists in India are utilising apps for neurological rehabilitation can help identify service gaps, enable more scalable interventions, and ultimately improve outcomes for both patients and practitioners. Therefore, this study aimed to investigate the utilisation of mobile applications by occupational therapists working in neurological rehabilitation in India. The following were the objectives of this present study-.

1. To determine the proportion and patterns of mobile app utilisation among Indian occupational therapists practising in neurorehabilitation.
2. To examine the use of mobile applications as tools for assessment and intervention in neurorehabilitation practice.
3. To explore the perceptions and attitudes of Indian occupational therapists toward mobile app utilisation in neurorehabilitation.

## Methods

### Study design

This study used a cross-sectional design to investigate the utilisation of mobile apps in neurorehabilitation practice among occupational therapists in India. India is a geographically vast and diverse country, and occupational therapists are spread across different regions. Considering the widespread distribution of occupational therapists across the country, we used an online survey using Google Forms to offer convenience and flexibility, ensure efficient and accurate data collection, and optimise research resources.

### Participants

The study participants were qualified occupational therapists who treated clients with neurological conditions and held a minimum Bachelor of Occupational Therapy qualification. Therapists were recruited irrespective of years of experience. Even early-career practitioners were considered in the study. We excluded occupational therapists who were using apps for nontherapeutic purposes and occupational therapy academicians not involved in clinical practice.

To determine the required sample size, a pilot survey of 10 occupational therapists was conducted to estimate the proportion (p) of occupational therapists working in neurological rehabilitation, as no prior data were available in the Indian context. The pilot results indicated that approximately 10% of OTs practice in this specialty (*p* = 0.10). Using this estimate, the initial sample size was calculated at the 95% confidence level (Z = 1.96) with a margin of error of 5% (d = 0.05). Using Cochrane’s formula for estimation, the initial sample size was calculated to be 135. Further, to account for a potential 20% non-response rate, the final sample size was adjusted to 169 participants to avoid underpowering the study.

### Instrument

An online survey questionnaire was employed to determine app utilisation. Approval to use and modify a survey questionnaire previously used in another study was obtained from the author [16] and is included in the supplementary file. It contained 28 questions, divided into five sections: demographic details; general app utilisation; app utilisation as an assessment; app utilisation as an intervention modality; reflections on app usage; and opinions on app usage based on their clinical experience. The content validity of the adapted questionnaire was established by five experienced occupational therapists with over 5 years of neurorehabilitation experience, who were selected to conduct the content validation. Using Lynn’s content validation method, experts rated the relevance of each item, and the content validity index (CVI) was calculated based on their agreement [28].

After obtaining consent from the subject experts, the content validation form was distributed online via Google Forms. Content validity was assessed using both the item-level CVI (I-CVI) and the scale-level CVI using the average method (S-CVI/Ave). Experts rated each item for relevance, and the I-CVI for each item was calculated as the proportion of experts who rated it 3 or 4 on the relevance scale. The overall S-CVI/Ave was 0.9, indicating that, on average, 90% of experts agreed on the relevance of the questionnaire items. This reflects excellent content validity, as values of 0.80 or higher are recommended by Polit and Beck [29] and Davis [30].

The final adapted survey comprised nineteen multiple-choice questions, allowing respondents to select multiple answers, four short-answer questions, and three open-ended questions. A skip logic feature was implemented for the general app usage section. Thus, participants who indicated that they did not use apps in this section were directed to the opinions section and could answer only the last three open-ended questions. Those who reported using apps were directed to answer the subsequent section on assessment and intervention practices. Similarly, the skip logic option was activated for the app usage section to complete either an assessment or an intervention. The reason for enabling the skip logic option was to allow participants using apps to complete the survey and ensure data reliability.

### Procedure

The study employed convenience and snowball sampling methods to recruit the participants. Even though it has limitations, these methods were chosen for their practicality and efficiency in reaching the participants. Ethical approval was obtained for this study, and all participants completed an online consent form before participation. Data were collected from November 2023 to February 2024.

A list of occupational therapists treating clients with neurological conditions was obtained from colleges, hospitals, rehabilitation centres, conferences, and individual occupational therapists. A concise introduction to the study, along with informed consent documentation, was sent via email or social media platforms, including Instagram, WhatsApp, and LinkedIn, to invite qualified occupational therapists working in neurorehabilitation to participate. A reminder alert was employed in this study to ensure maximum participation and minimise non-response bias. Four reminders were sent to non-responsive participants at one-week intervals.

### Data analyses

The responses from the Google Sheet were downloaded, screened, and coded in Microsoft Excel before being transferred to Jamovi 2.3.28. Demographic characteristics were analysed using descriptive statistics. Additionally, three open-ended questions were coded and analysed using a summative content analysis approach [31]. This method combines quantitative frequency analysis with qualitative interpretation to provide both descriptive and interpretive insights. Initially, the textual data were reviewed, and the relevant codes were generated. The frequency of each code was then quantified to identify the predominant categories and patterns within the data. The resulting categories were organised according to the components of the HAAT model to ensure a structured and theory-driven interpretation.

## Results

### Demographic information of the participants

A total of 169 occupational therapists participated in the survey. Three responses were excluded due to incomplete data, leaving 166 occupational therapists in the final analysis. The descriptive statistics indicate that participants reported a range of experience in neurorehabilitation, from 1 month to 28 years. The median experience was 2 years, with an interquartile range (IQR) of 3.8 years. As shown in Table 1, most participants (57.2%) reported that their highest qualification was a bachelor’s degree. A large proportion, 91% of occupational therapists, were working as clinical practitioners, meaning they were fully engaged in treating clients with neurological conditions. Most participants worked within a hospital-based setting, accounting for 60.2% of the respondents.

**Table 1:** Demographic characteristics of the participants

Demographic characteristics		*n* (%)	Participants app usage	
			User(*n*)	Non-user (*n*)
**Qualification**				
	Bachelors	95 (57.2%)	39	56
	Masters	68 (41%)	29	39
	Doctorate	3 (1.8%)	2	1
**Current designation**				
	Practitioner	151 (91%)	63	88
	Both practitioner and academician	15 (9%)	7	8
**Work Setting**				
	Hospital	100 (60.2%)	43	57
	Rehabilitation center	52 (31.3%)	23	29
	Others*	14 (8.4%)	4	10
**State**				
	Tamil Nadu	78 (47%)	31	47
	Kerala	28 (16.9%)	8	20
	Karnataka	18 (10.8%)	10	8
	Maharashtra	22 (13.3%)	12	10
	Others**	20 (12%)	9	11

* Community based 5(3%), Home health 3(1.8%), Institution 5(3%) and Online consultation 1(0.6%)

** Telangana 1(0.6%), Orissa 1(0.6%), New Delhi 4(2.4%), Madhya Pradesh 2(1.2%), Andhra Pradesh 1(0.6%), Goa 3(1.8%), Rajasthan 1(0.6%), Gujarat 2(1.2%), Meghalaya 3(1.8%) and Bihar 1(0.6%)

### App utilisation by occupational therapists in clinical practice

Of the 166 occupational therapists included in the analysis, 70 (42%) reported using apps in their clinical practice. Among therapists who used apps (*n* = 70), the primary target population was adult neurological clients.

### App utilisation as an assessment by occupational therapists in clinical practice

Among the 70 occupational therapists who reported using apps in clinical practice, 28 (40%) used them for assessment purposes. The most frequently assessed client factors were cognition (*n* = 26, 92.9%), motor function (*n* = 17, 60.7%), hand function (*n* = 17, 60.7%), perceptual function (*n* = 17, 60.7%), balance (*n* = 15, 53.6%), coordination (*n* = 14, 50%), sensory function (*n* = 8, 28.6%), and level of consciousness (*n* = 7, 25%). Additionally, 13 therapists (46.4%) reported using apps to assess language. Occupational performance areas assessed included ADL (*n* = 9, 32.1%), Instrumental ADL (*n* = 8, 28.6%), and Leisure (*n* = 7, 25%). The top three apps clinicians identified as most often used during the assessment were Cognifit, Dexteria, and Neuroforma, as described in Table 2, categorised by the domains of the Occupational Therapy Practice Framework (OTPF-4) [19]. Additionally, 10 occupational therapists (35.7%) reported having received training in using apps for clinical practice. Workplace support for using apps in assessments was reported by 24 (85.7%) participants. The devices most commonly used for app-based assessments were smartphones (46.4%), laptops (21%), and computers (21%).

**Table 2:** Distribution of apps used as an assessment in practice

Domain	List of apps used as an assessment (*n*)
Mental functions	Cognifit (5), Boston Cognitive Assessment (1), Psychopy (1), Lumosity (1), Brain Maze (1), Focus (1), Neuronation (1), Brain Games (1), Sudoku (1), Wordplay (1), and Pictoword (1)
Neuromusculoskeletal and movement-related functions	Neuroforma (2), Balzepod (1), Fitmi (1), Armeo senso (1), Armable (1), Angulus (1), Rewin Health (1), REBA (1), GMFM (1), Dexteria (2), Armeo power (1), Hand Tutor (1), Bobo balance (1), Balance board (1), and Typing master (1)
Sensory function	ISNCSI Algorithm (1)
Occupation (ADL)	Toothbrush Reminder (*n* = 1)
Others	Google Forms (1), Vikas (1), Insight timer (1), Google Meet (1), Nuessabaum (1), Fun bubbles (1), Ludo (1), VR (1), and Pluto (1)

### App utilisation as an intervention by occupational therapists in clinical practice

Among the 70 occupational therapists, 56 (80%) reported using apps as part of their interventions. Most participants reported using apps to address deficits in cognition (*n* = 52, 92.9%), hand function (*n* = 28, 50%), motor function (*n* = 25, 44.6%), coordination (*n* = 26, 46.4%), perceptual function (*n* = 24, 42.9%), balance (*n* = 21, 37.5%), consciousness (*n* = 8, 14.3%), sensory function (*n* = 4, 7.1%), language (*n* = 16, 28.6%) and other areas (*n* = 1, 1.8%). Mobile apps used for occupational performance interventions were most commonly applied in ADL (*n* = 15, 26.8%), followed by Leisure (*n* = 10, 17.9%) and IADL (*n* = 6, 10.7%). The top three apps clinicians identified as most often used as treatment modalities were Cognifit, Lumosity, and Neuroforma, as shown in Table 3. Regarding the use of apps as a treatment modality, 21 participants (37.5%) reported having received formal training in this area. Furthermore, 50 participants (89.3%) reported that their workplace provided support for integrating apps into therapeutic practice. Among the devices used for app-based interventions, smartphones (36%) and tablets (30%) were the most frequently used.

**Table 3:** Distribution of apps used as a treatment modality in practice

Domain	List of apps used as a treatment modality (*n*)
Mental functions	Cognifit (14), Boston Cognitive Assessment (1), Pyschopy (1), Lumosity (6), Brain maze (1), Focus (1), Neuronation (1), Cognizant (1), Neurobics (1), Visuospatial (2), Spot the difference (1), Blocks game (1), Brain gym (1), Train your brain (2), To-do-list (1), Brain fitness Pro (1), Mind maze (1), Pictoword (1), Elevate (1), Wordscape (1), Mindpal (1), and Picture matching (1)
Neuromusculoskeletal and movement-related functions	Neuroforma (3), Balzepod (2), Fitmi (2), Armeo senso (1), Armable (1), Angulus (1), Rewin Health (1), REBA (1), Motor Imagery (1), Armeo Power (1), Mind motion go (1), Boho motion (1), Music glove (1), Saebo Rejoyce (1), Dexteria (2), Hand tutor (2), Typing Master (1), Bobo balance (1), Balance master (2), and Balance board (2)
Sensory function	Metronome (1) and Awaz (1)
Others	VR (2), Fun bubbles (1), Skillz (1), Ludo (1), Samsung Game launcher (2), Youtube (2), Spotify (1), Pluto (2), Meditutor (1), Vikas (1), Midland (1), Tactus (1), Insight Timer (1), Medbridge (1), Google meet (1), My health manager (1), and Physitrack (1)

### Reflections on app usage among occupational therapists

Occupational therapists reported the primary reasons for app use, the effectiveness of apps, whether they would recommend apps to others, whether they would participate in continuing education, and whether they would be involved in the development of mobile applications. Participants who used apps identified client engagement (34%) and convenience/ease of use (23%) as the most common reasons for using apps in practice. Similarly, 38 (54.3%) of these participants reported that the effectiveness of using apps as an intervention modality was comparable to that of traditional methods. In comparison, 32 (42.7%) reported that it was better than conventional methods. Additionally, 50 (71.4%) reported recommending apps to other healthcare professionals. Most occupational therapists reported that they would participate in continuing education about using apps (91.4%) and involvement in the development of new apps (97.1%) in clinical practice.

### Facilitators, barriers, and future recommendations for app usage

Participants’ perceptions of facilitators, barriers, and future recommendations for app use were analysed using the components of the HAAT model. The first section describes the quantitative analysis using the rank, categories, code count, and elements of the HAAT model. Furthermore, the second section employs a thematic analysis approach, focusing on categories, theme groups, and verbatim statements from occupational therapists regarding facilitators, barriers, and recommendations for mobile app use. Data related to this section, including the participant verbatims, are provided in the supplementary file. The first section describes the quantitative analysis using rank, categories, code counts, and the HAAT model elements, as presented in Table 4.

**Table 4:** Facilitators, barriers, and future recommendations for app usage

**Rank**	**Categories**	**Code count**	**Component of the HAAT Model**
**1) Advantages/Facilitators of app use in practice**			
1	User-centric and versatile apps	95	Assistive Technology
2	Engagement of the client in activities	42	Activity
3	Improving the client’s well-being	36	Human
4	Efficiency and Effectiveness of the apps	33	Assistive Technology
5	Enhanced quality of care and data management through apps	29	Assistive Technology
**2) Disadvantages/Barriers of app use in practice**			
1	Non-usability and practicality of apps	66	Assistive Technology
2	Professional conduct and clinical practices of apps	64	Assistive Technology
3	Limited personal factors for app usage	56	Human
4	Cost and financial considerations of the app	26	Assistive Technology
5	Contextual factors influencing app usage	9	Context
**3) Future recommendations for app use in practice**			
1	User-centric and domain-specific apps in clinical practice	107	Assistive Technology
2	Enhancing client outcomes	42	Human
3	Incorporation of OT process and care in app-based practice	37	Assistive Technology
4	Enhancing collaboration in app-based practice	11	Activity
5	Cost and efficiency of apps	8	Assistive Technology

### Diversity of perspectives related to the use of mobile apps

#### ***Theme 1***: ***Viewing app use through the human perspective***

From a human perspective, participants reported that app use facilitated clients’ well-being, leading to improvements in cognition, knowledge, and skill development. However, barriers to app utilisation stemmed from personal factors, including limited knowledge, technological unfamiliarity, and addiction. Optimising future app usage involves prioritising enhancements to client outcomes, encompassing improved well-being, knowledge acquisition, heightened awareness, and comprehensive training.

#### ***Theme 2: Viewing app use through the perspective of activity***

Viewing the client’s engagement in activities through the lens of activity, comprising active participation, motivation, and feedback, emerges as the primary facilitator of change while using apps. Additionally, enhancing collaboration in app-based practice, including teamwork and effectiveness in conjunction with conventional therapy, is a crucial recommendation for the future.

#### ***Theme 3: Viewing app use through the lens of technology***

From an assistive technology perspective, occupational therapists prioritised user-centric, versatile apps above all else. Efficiency, effectiveness, quality, and data management of apps were other key factors that facilitated app usage in this sample. However, they also identify several barriers, including concerns about professional conduct and clinical practices, usability and practicality issues, and cost and financial considerations associated with app use. Looking ahead, recommendations for future app-based practices include focusing on user-centric and domain-specific apps to better meet patients’ needs. Emphasising the integration of occupational therapy processes and ensuring the quality of care provided through these apps were deemed important. Moreover, participants considered that attention to cost-effectiveness and efficiency will be essential for widespread adoption and sustainability of mobile apps in occupational therapy practice.

#### ***Theme 4: Viewing app use through a contextual perspective***

Considering the context, the participants identified cultural differences and socialisation as significant barriers to app usage. These contextual factors created hurdles that they felt impeded the seamless integration of apps in practice.

## Discussion

This cross-sectional study aimed to explore the use of apps in neurological rehabilitation practice among occupational therapists in India. The study results suggest that less than half of participants use apps with clients with neurological conditions, suggesting that not all occupational therapists integrate apps into clinical practice. A similar finding in the study by Seifert et al. [25] states that 47% of Ohio-based occupational therapists used the apps in practice compared to 71.9% in a nationwide survey. The lower adoption of app use by occupational therapists in clinical practice may be due to a lack of knowledge, inadequate training, the absence of evidence-based apps, cost, and unfamiliarity [25, 32, 33].

Occupational therapists in the present study predominantly reported using apps in hospital settings, particularly with clients aged 18–60 years. This contrasts with findings from Seifert et al. [25] and Davis-Cheshire et al. [16], who reported that apps are more commonly utilised in pediatric, school-based, and early intervention settings. However, it is worth noting that Davis-Cheshire et al. (2020) acknowledged that their sample included a higher proportion of therapists working in school settings, which may explain this difference. In the current study, most participants used apps primarily as a treatment method (80%) rather than for assessment (40%) in clients with neurological conditions. This trend aligns with the findings of Davis-Cheshire et al. (2020), who reported that 84.3% of therapists used apps for treatment, while only 19.1% used them for assessment.

The comparatively lower use of apps for assessment observed in the present study and in Davis-Cheshire et al. (2020) suggests a possible limitation in the availability or suitability of assessment apps. Assessments that require specific tools are likely to be more challenging to adapt to an app-based format. They may still need traditional paper forms for evaluation, whereas those focused on client self-reports are more easily adapted to app-based platforms.

In this study, cognition emerged as a primary impairment that practitioners target when using apps for evaluation and intervention. Gentry et al. [32, 33] support that apps act as compensatory mechanisms for cognitive deficits. The realm of cognitive rehabilitation for cognitive dysfunction resulting from neurological disorders is steadily growing within neurological rehabilitation [34]. This area holds high significance and is frequently undertaken by occupational therapists as a core responsibility during rehabilitation [35].

Participants identified CogniFit as the most common assessment (*n* = 5) and treatment modality (*n* = 14). Yaneva et al. [36] found that CogniFit’s General Cognitive Assessment Battery (GCAB) is promising for assessing cognitive function in older Bulgarian adults, indicating it could replace traditional neuropsychological batteries due to its strong internal consistency and concurrent validity. According to LeBeau et al. [17], Lumosity ranked fourth among the top 25 mobile applications evaluated using the Mobile Application Rating Scale (uMARS), demonstrating high quality across domains such as engagement, functionality, and aesthetics, with an average overall rating of 4.718 out of 5. The present study identified Lumosity as the second-most-frequently used app for treatment by clinicians due to its high quality.

In addition to cognitive functions, participants in this study also utilized apps to assess and treat hand and motor skills in patients with neurological disorders. According to Sawant et al. [22], the Dexteria app provides repetitive app-based therapy that may benefit fine motor function in individuals with stroke, suggesting that digital platforms can support structured practice to improve motor control. Through touchscreen interfaces, patients engage in task-oriented training, developing skills that enable them to perform real-world tasks seamlessly [37].

Therapists in our study who have incorporated apps into their occupational therapy practice have identified several factors influencing their use. The primary reasons, as perceived by the respondents, are engagement and ease of use. Similar findings of Davis-Cheshire et al. [16] suggest that occupational therapists and occupational therapy assistants reported engagement as the main reason for app usage among United States occupational therapists. Apps offer several advantages in clinical practice. The accessibility and portability of apps enable extensive repetition, which is essential for motor learning, while also providing an engaging balance of fun and challenge for patients [22].

Regarding the effectiveness of apps as an intervention modality, all participants in the present study (100%) perceived apps as at least as effective as traditional interventions. This perception is consistent with the findings of Davis-Cheshire et al. [16], who reported that 96.4% of occupational therapy practitioners rated apps as comparable to or superior to conventional therapeutic approaches. Together, these findings indicate a growing acceptance of mobile apps as credible intervention tools in occupational therapy practice.

However, the use of apps for both assessment and intervention requires therapists to possess adequate technological and clinical competence [16]. Owning a personal app-based device does not necessarily mean possessing the required skills and competencies to use it effectively as an intervention tool [38]. Therefore, practitioners are advised to seek training before incorporating app-based devices into their practice [8]. Developing competence in the use of mobile app-based interventions is essential to uphold practitioners’ ethical obligations, as it ensures safe, evidence-based, and client-centred care. Adequate digital proficiency enables therapists to select appropriate applications, maintain data privacy, and use technology responsibly, thereby promoting both practical and ethical therapeutic outcomes [39, 40].

The present study also explored occupational therapists’ opinions on app use, focusing on facilitators, barriers, and future recommendations using the HAAT model. From the HAAT model’s human perspective, occupational therapists identified enhancing client well-being as a critical factor in app use in practice. They emphasised that apps are particularly effective in improving cognition, motor function, and hand function, thereby significantly enhancing participation and well-being for clients with neurological conditions.

Notable challenges included varying levels of client knowledge about technology use and mobile device addiction, which were barriers yet to be effectively addressed in app-based practice. The barriers identified in this study align with those identified in previous research [41]. Participants who are familiar with mobile devices often trust technology and usually rely on their technical skills [42]. Similarly, the need for knowledge and training in technology use presents a significant hurdle [43]. These findings emphasize the need for improving client knowledge and therapist training to enhance the effectiveness of app-based interventions in occupational therapy.

From an activity perspective, engagement was identified as a significant facilitator for app usage, echoing its importance as a participant’s primary reason for integrating apps into practice, as previously mentioned. Additionally, participants recommended incorporating teamwork and aligning app use with conventional therapy in future practice. Sawant et al. [22] found that combining app-based treatment with conventional methods improved functional hand recovery in stroke patients. These findings suggest that integrating app-based interventions with conventional therapy may enhance rehabilitation outcomes.

Cultural differences and a lack of socialisation emerged as notable barriers for participants from the HAAT model’s context perspective. Erickson [42] also suggested that understanding cultural priorities regarding device usage in daily occupations is crucial. In the Indian context, understanding the client’s cultural and personal background is essential, as social attitudes, family involvement, and community support systems strongly influence the acceptance and utilisation of mobile technologies in rehabilitation.

There is a need for more evidence to address concerns about the quality and safety of apps and mobile devices. In the previous studies, barriers included the overwhelming number of existing apps, lack of customisation options, and payment requirements for full app functionality [40–42]. There is a need for more evidence to address concerns about the quality and safety of apps and mobile devices in clinical practice [43]. Cost-related issues, including the expense of products and device usage, impede adoption [22]. Erickson [42] emphasized the importance of considering financial resources for device access, technologies, and internet availability, emphasizing their impact on app usage in practice.

Participants in our study recommended that future app development prioritise user-centric and domain-specific features, incorporating key occupational therapy processes while maintaining affordability and efficiency in clinical practice. This aligns with previous studies [44, 45] that reported that maintaining client confidentiality is crucial and that there is a growing need for personalised app solutions tailored to individual stroke survivors and their support networks, enabling them to engage actively in rehabilitation.

This study demonstrated greater awareness of the use of apps in clinical practice, as well as the importance of app-based devices among occupational therapists. Future research involving apps in assessment and intervention will encourage occupational therapists to utilise them with clients undergoing neurological rehabilitation. Additionally, involving occupational therapy practitioners in app design ensures a focus on addressing clients’ occupational performance needs. This may facilitate the efficient utilisation of apps with clients who have neurological conditions, thereby improving outcomes and enhancing the quality of occupational therapy services.

While the findings of this study provide valuable preliminary insights, they should be interpreted with caution due to the limited sample size, which may not fully represent occupational therapists working with clients with neurological conditions across India. The reliance on self-reported data and voluntary participation may also introduce self-selection bias, and the survey tool, developed specifically for this study, lacked established psychometric validation.

Despite these limitations, the findings highlight the growing relevance of app-based interventions in neurological rehabilitation and underscore the need for structured training programs to enhance therapists’ digital competencies. From a policy perspective, integrating digital health literacy and mobile applications into professional development frameworks and rehabilitation guidelines could strengthen evidence-based practice. Future research should focus on large-scale, multi-site studies using psychometrically robust tools to explore the efficacy, accessibility, and implementation of app-based interventions in India and other resource-limited contexts.

## Conclusion

Occupational therapists in India use apps widely in neurological rehabilitation, primarily for treatment focusing on cognition, but face barriers such as a lack of knowledge, training, evidence-based apps, and cost considerations. Despite challenges such as varying client knowledge and cultural diversity, therapists perceive apps as effective or superior to traditional methods, emphasising engagement, ease of use, and perceived usefulness. This suggests that addressing these barriers and enhancing facilitators can maximise the potential of app-based interventions in neurological rehabilitation.

## Supplementary Information

Below is the link to the electronic supplementary material.

Supplementary Material 1

Supplementary Material 2

## Data Availability

The research data can be made available on reasonable request and/or after all related publications are concluded.

## Abbreviations

OTs: Occupational Therapists
HAAT: Human Activity Assistive Technology Model
