# Supplementary material for: Utilisation of mobile apps in neurological rehabilitation practice among occupational therapists in India: a cross-sectional survey
Source: BMC Health Serv Res. 2026 Feb 3;26:238. doi: 10.1186/s12913-026-14098-w (PMC12903628; doi:10.1186/s12913-026-14098-w)
Supplement: Supplementary file 1 — Supplementary Material 1 [file 12913_2026_14098_MOESM1_ESM.docx]

**Supplementary Table 1**

*Opinions of occupational therapists on facilitators of app usage*

| **Rank** | **Categories** | **Theme** | **Verbatim of the participant's responses** |
| --- | --- | --- | --- |
| 1 | User-centric and versatile apps | Assistive Technology | - “Apps will make data easily accessible and recorded automatically. Easy to use with today's generation's dependency on mobile and the internet” (P128) - “Using apps, the therapy can be more creative and will not be monotonous. Patients will be more interested, and home programs can be customized and personalized easily” (P94) - “East to carry the phone or tablet, it can be used anywhere in any situation” (P142) |
| 2 | Engagement of the client in activities | Activity | - “Apps are easily approachable to clients to make them engaged in activity” (P156) - “Many apps are designed with interactive features, making therapy sessions more engaging and motivating for clients, especially children or individuals with cognitive impairments” (P123) - “Enhanced motivation - due to the immersive nature of apps, increased motivation is noted; Engaging experience, encouraging consistent participation and adherence to therapy plans; Easy simulation of real-world environments and promotion of neuroplasticity; Instant feedback and easy-to-monitor progress; Safe environment - the client can practice meal preparation skills without the use of fire/sharp objects” (P87) |
| 3 | Improving the client's well-being | Human | - “It was helpful when I was treating patients in a cognitive aspect, but it also improved other skills and capabilities. Helps to give or work on so much of components in less time and helps to convey to caregivers how much the OT can help in the rehabilitation process in all possible ways” (P67) - “Enhancing the quality of treatment and improvement in health care” (P32) - “It will easily improve patients' functional skills with interest” (P62) |
| 4 | Efficiency and Effectiveness of the apps | Assistive Technology | - “Save a lot of time, energy, and money without compromising on patient/client health and progress. Beneficial for both patients and therapist” (P110) - “Provides variation in the treatment, easy accessibility, convenient for pt as well as he/she can use it even when a therapist is not around” (P141) - “Using apps as an assessment and intervention will be more accurate than the traditional method, saves time, helps the patient to be engaged in an activity for a longer time, which eventually helps with their faster recovery (better progress) and gives constant feedback” (P4) |
| 5 | Enhanced quality of care and data management through apps | Assistive Technology | - “Can be easy to develop goals and track them based on clients progress; Easy to maintain records” (P129) - “It becomes accessible for documentation, collection of data, retrieval of stored information regarding the patient or sessions “(P3) - “Evidence shows that it's beneficial for clients to engage in these apps as they are exciting, which gives a good prognosis in their performance areas” (p 28) |

| **Rank** | **Categories** | **Theme** | **Verbatim of the participant's responses** |
| --- | --- | --- | --- |
| 1 | Non-usability and practicality of apps | Assistive Technology | - “Sometimes we may not find the proper app as per the patient's requirements, we have to use available game apps for patient treatment which sometimes may not meet all the therapeutic requirements” (P58) - “The only barrier I faced is: Most of the patients from rural areas doesn't have a smartphone to assess such apps” (P147) - “Not feasible for clients with visual-perceptual issues, not all clients are tech-oriented, connectivity issues may hinder the use of internet-based apps, in-app payments, lack of training from institutions and professional governing bodies regarding the use of apps in clinical practice” (P47) |
| 2 | Professional conduct and clinical practices of apps | Assistive Technology | - “It may not be suitable for all types of patients we come across; some might be uneducated or unfamiliar with the new age technologies. and as a therapist, I don't prefer or believe an AI or other apps to give therapy compared with hands-on experience of a therapist” (P28) - “Sometimes the therapy explained has to be more practical and also most are not evidence-based.” (P5) - “Though it is useful, it can't apply to all neurological cases, and patients should have basic cognition to apply it and understand the concepts. Therapists should have a proper idea about the application of apps and their uses. Can't expect an outcome while practicing ...it might be tricky for the therapist to predict the prognosis, sometimes it seems to be more unrealistic” (P38) |
| 3 | Limited personal factors for app usage | Human | - “Not all patients have access to smartphones. Among the ones that do have a smartphone, not everyone has the know-how to use certain apps due to language barriers and knowledge levels (P54) - For old age people and who are illiterate people, which does not apply to implementing the app-based intervention, and the client was not interested in participating in app-based practice (P13) - It might make the patient recovery very slow, and the patient will be dependent on the apps more than their skill” (P102) |
| 4 | Cost and financial considerations of the app | Assistive Technology | - “Costly, not accessible for everyone, institutions are not ready to invest” (P164) - “The disadvantage is that each application is paid and serves only one component. So if we have a common application which helps in Motor, Sensory function, and Cognitive it will be well and good to serve the community” (P91) - “Expensive and not user friendly” (P36) |
| 5 | Contextual factors influencing app usage | Context | - “Compliance by the client to use it religiously and technical issues” P(120) - “Disadvantage - Man is a social being; in terms of Paediatrics-- Child development groomed through social situations!!.. And again, generally, now most people are coping with their stress using digital platforms where the underlying SOCIALLY VACANT SPACE for stress is least recognized and understood by themselves!!... Felt as Therapist must aware of SOCIAL HEALTH too when determining as Holistic approach in OT “(P85) |

**Supplementary Table 2**

*Opinions of occupational therapists on barriers to app usage*

| **Rank** | **Categories** | **Theme** | **Verbatim of the participant's responses** |
| --- | --- | --- | --- |
| 1 | User-centric and domain-specific apps in clinical practice | Assistive Technology | - “We can implement app-based practices which are being created for specific neurological conditions, and it has to be validated through the proper longitudinal study” (P67) - “App should have all the domains required for assessment and intervention. Apps should be easily accessible and simple” (P129) - “Apps should be user friendly, intuitive, should maintain confidentiality” (P129) - “To promote awareness and call OT volunteers to work on App-based practice to make it a universal, unbiased one” (P110) |
| 2 | Enhancing client outcomes | Human | - “App-based practice is one of the ways which can make us unique in what we do, and also it may enhance the client's factors and functions” (P151) - “Use apps only when you are confident that the patients will benefit and also use the right apps” (P78) - “Would be beneficial if teaching institutions or professional governing bodies compiled essential app-based resources as well as provided training for occupational therapists working in the field of neurorehabilitation” (P47) |
| 3 | Incorporation of OT process and care in app-based practice | Assistive Technology | - “An app to track patients' activities or check activity compliance might be beneficial; apps can focus on covering broader aspects of a client's problem. Apps also should have parameters to record the client's progress. Apps should be made accessible to all population and should be made easy to use so that the caregivers can use it at home as well” (P54) - “It would be great if the components were kept simple so anyone could easily understand them. And different components can be addressed and grading those based on activities from simple to complex will be very helpful” (P66) - “Adequate training and user-friendly interface, increasing the repertoire of activities including simulating ADL, selecting the appropriate client and making an appropriate treatment plan” (P51) |
| 4 | Enhancing collaboration in app-based practice | Activity | - “Please make sure everything in the rehab team is aware of app-based interventions and do not talk about it in a less than positive way in front of the patients” (P26) - “OTs should be open to new technologies, including apps and virtual rehabilitation. Apps cannot replace traditional methods but can add value to therapy. Due to extensive knowledge of activity analysis, OTs are the right professionals to take part in the development of apps that mimic real-life scenarios and improve participation in ADLs” (P87) |
| 5 | Cost and efficiency of apps | Assistive Technology | - “Can be more economically efficient” (P38) - “Easy to understand, video explanations of the app and activities and links to buy a product if required in the activity for process” (P159) |

**Supplementary Table 3**

*Opinions of occupational therapists on future recommendations of app usage*
