# Supplementary material for: Utilisation of mobile apps in neurological rehabilitation practice among occupational therapists in India: a cross-sectional survey
Source: BMC Health Serv Res. 2026 Feb 3;26:238. doi: 10.1186/s12913-026-14098-w (PMC12903628; doi:10.1186/s12913-026-14098-w)
Supplement: Supplementary file 2 — Supplementary Material 2 [file 12913_2026_14098_MOESM2_ESM.docx]

**Questionnaire**

1. What is your highest degree earned in Occupational Therapy?
   1. Bachelors
   2. Masters
   3. Doctorate
2. How many years have you been practicing as an occupational therapist?
3. Which is your current designation? (Ex: Clinical therapist, Supervisor, Head of department, etc..,)
4. Country:
5. State:
6. Type of working setup:
   1. Hospital
   2. Rehabilitation centre
   3. Home Health
   4. Community-based
   5. Others (Please Specify)
7. Have you ever used apps in Neurological Occupational therapy practice?
   1. Yes
   2. No
8. Over the course of your career, what is the primary population with which you have used apps?
   1. Paediatric Neuro Population
   2. Adult Neuro Population
   3. Geriatric Neuro Population
9. Have you used apps during the assessment?
   1. Yes
   2. No
10. Over the course of your career, which client impairments have you used apps to address? (Check all that apply)
    1. Cognition – Attention, Memory, Orientation, Executive function
    2. Consciousness
    3. Language
    4. Perceptual skills
    5. Motor functions
    6. Hand functions
    7. Sensory function
    8. Balance
    9. Coordination
    10. ADL
    11. IADL
    12. Leisure
    13. Others (please specify)
11. List out the name of apps used in the assessment process with their availability (Ex: App name - Free/Paid)
12. Have you ever undergone any training for using apps in the assessment process?
    1. Yes
    2. No
13. What is the most app-based device you have used in practice for the assessment?
    1. Smartphone
    2. Tablet
    3. Laptop
    4. Other (please specify)
14. Have you ever undergone any training for using apps in the assessment process?
    1. Yes
    2. No
15. Have you used apps as an intervention?
    1. Yes
    2. No
16. Over the course of your career, which client impairments and occupations have you used apps to intervention? (Check all that apply)
    1. Cognition – Attention, Memory, Orientation, Executive function
    2. Consciousness
    3. Language
    4. Perceptual skills
    5. Motor functions
    6. Hand functions
    7. Sensory function
    8. Balance
    9. Coordination
    10. ADL
    11. IADL
    12. Leisure
    13. Others (please specify)
17. List out the name of apps used as modalities during treatment with their availability (Ex: App name - Free/Paid)
18. Have you ever undergone any training for using apps as modalities during treatment?
    1. Yes
    2. No
19. What is the app-based device you have used most in the intervention process?
    1. Smartphone
    2. Tablet
    3. Laptop
    4. Other (please specify)
20. Does your workplace support the use of apps as modalities during treatment?
    1. Yes
    2. No
21. What is the primary reason you have used apps in practice?
    1. Convenience/ease-of-use
    2. Portability
    3. Perceived effectiveness
    4. Versatility
    5. Engagement
    6. Other (please specify)
22. Based on your experience, how would you rate the effectiveness of apps as an intervention modality?
    1. Better than traditional methods
    2. About the same as traditional methods
    3. Worse than traditional methods
23. Have you ever recommended apps to healthcare professionals for use with clients?
    1. Yes
    2. No
24. Would you pursue continuing education about the use of apps in occupational therapy?
    1. Yes
    2. No
25. Are you interested in participating in the development of occupational therapy apps?
    1. Yes
    2. No
26. Based on your experience, please comment the advantages/facilitators of apps in practice
27. Based on your experience, please comment the disadvantages/barriers of apps in practice
28. As an OT what are the recommendations, would you like to give in an app based practice in Neurological rehabilitation?
